# OD-seq: outlier detection in multiple sequence alignments

**DOI:** 10.1186/s12859-015-0702-1

**Published:** 2015-08-25

**Authors:** Peter Jehl, Fabian Sievers, Desmond G. Higgins

**Affiliations:** UCD Conway Institute of Biomolecular and Biomedical Sciences, University College Dublin, Dublin, 4 Ireland

**Keywords:** Outlier, Multiple sequence alignment

## Abstract

**Background:**

Multiple sequence alignments (MSA) are widely used in sequence analysis for a variety of tasks. Outlier sequences can make downstream analyses unreliable or make the alignments less accurate while they are being constructed. This paper describes a simple method for automatically detecting outliers and accompanying software called OD-seq. It is based on finding sequences whose average distance to the rest of the sequences in a dataset, is anomalous.

**Results:**

The software can take a MSA, distance matrix or set of unaligned sequences as input. Outlier sequences are found by examining the average distance of each sequence to the rest. Anomalous average distances are then found using the interquartile range of the distribution of average distances or by bootstrapping them. The complexity of any analysis of a distance matrix is normally at least *O*(*N* ^2^) for *N* sequences. This is prohibitive for large *N* but is reduced here by using the mBed algorithm from Clustal Omega. This reduces the complexity to *O*(*N* log(*N*)) which makes even very large alignments easy to analyse on a single core. We tested the ability of OD-seq to detect outliers using artificial test cases of sequences from Pfam families, seeded with sequences from other Pfam families. Using a MSA as input, OD-seq is able to detect outliers with very high sensitivity and specificity.

**Conclusion:**

OD-seq is a practical and simple method to detect outliers in MSAs. It can also detect outliers in sets of unaligned sequences, but with reduced accuracy. For medium sized alignments, of a few thousand sequences, it can detect outliers in a few seconds. Software available as http://www.bioinf.ucd.ie/download/od-seq.tar.gz.

## Background

Multiple sequence alignments are essential for many sequence analysis tasks [1–4]. They are widely used for phylogenetic analysis, discovery of conserved regions, protein function studies and structure prediction. One problem that can arise in these analyses is the presence of outlier sequences. Outliers can disrupt an alignment at the construction stage and lead to highly sub-optimal alignments with a knock-on effect on downstream analyses. With small datasets, these can often be seen in the final alignment, if a viewer such as Jalview [5] is used. With very large alignments of say 10 s of thousands of sequences, it can be hard to view the complete alignment and it can be difficult to see outliers.

Outliers can come from a variety of sources. The simplest example is a sequence that is not homologous with the rest of the dataset and which has been included by accident. These are the easiest to detect as they will not have any of the conserved regions which the rest of the sequences might share. The pattern of gaps and conserved blocks will be completely different between the outlier and the rest. A second source of outliers will be sequences which have been partly mistranslated due to a sequencing error or incorrect automated translation from a genome sequence. These will have some conserved blocks followed by sections that are non-homologous with the rest. The longer the mistranslated region, the easier it will be to detect but very short outlier sections may be hard to find. The third kind of outlier will be where a sequence is homologous to the rest but where the similarity is extremely low and it is very hard to align. This case is a matter of choice and of degree. Some data sets need to include all possible homologues, for completeness. Others need to restrict dataset membership to sequences which are alignable over their full length. This case is different to the first two because very distant sequences may be hard to distinguish from real outliers which are simply not homologous.

Outlier detection is a well studied field in computer science and has been applied to many different fields such as credit card, insurance and tax fraud detection, intrusion detection for cyber security, fault detection in safety critical systems, military surveillance for enemy activities and many other areas [6]. Previous attempts at detecting sequence outliers have mainly concentrated on finding sections of sequence or alignment which are highly divergent. In Clustal X [7] there are menu items to activate a variety of outlier detection schemes which are based on detecting runs of amino acids with suspiciously low similarity scores to the rest. These can then be highlighted in various ways for visual detection. The GBLOCKS package [8] finds sections of alignment that are rich in gaps and which have low overall conservation. These blocks can be automatically removed for automated creation of phylogenies in pipelines. Penn et al. [9] used robustness to guide tree changes as a measure of alignment confidence. The DivA package [10] uses training sets seeded with manually inserted problem segments to recognise outlier positions or segments.

In this paper, we introduce the OD-Seq package which attempts to identify outlier sequences in a multiple alignment. It uses a simple gap based metric which counts the number of positions between two aligned sequences which have a gap in one and not the other. These distances are used to make a distance matrix which is then analysed to find sequences with unusually high average distances from the rest of the sequences. We can also use OD-Seq to find outliers in sets of unaligned sequences using pairwise BLAST [11] scores but this is less sensitive. We demonstrate the use of OD-Seq by taking sets of sequences from Pfam [12] and seeding these with outlier sequences from other Pfam families. In principle our method should work for nucleic acid sequences as well, but hasn’t been tested.

## Methods

### Algorithm

#### Overview

**Fig. 1:**
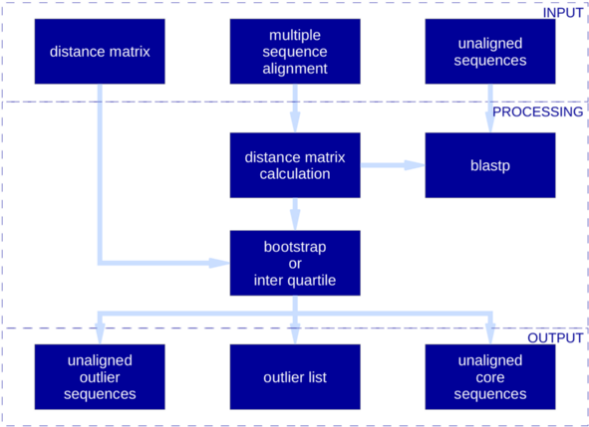
Software flow chart. Infographic illustrating the workflow of OD-seq. At the input layer the user can provide a multiple sequence alignment, unaligned sequences or a pre-computed distance matrix. The sequence files are used to compute a distance matrix. This data are then normalised by bootstrap or interquartile range analysis. Outlier list, core sequences and outlier sequences are available for output

The original motivation for designing OD-seq was to find outlier sequences in large multiple sequence alignments. This is the ideal usage for the program but it can also be used to detect outliers in sets of unaligned sequences. The suggested workflow would be to compute an alignment first, followed by using OD-seq to remove outliers and realigning the truncated set of sequences to obtain the final alignment. The basis of the algorithm is to analyse a matrix of gap-based distances between a set of sequences. Then outliers are found by looking for sequences with unusual average distances to the remainder of the sequences. This methodology will not work if excessively heavy gap penalties are imposed as only few gaps would be present in the MSA, considerably reducing the power of the present approach. In general, accurate gap placement is a difficult problem [13]. The OD-seq algorithm is divided in two main parts. First we create or take as input the distance matrix. Then we analyse the vector of mean distances from each sequence to the rest to predict outliers (Fig. 1). Different pairwise distance metrics are used for aligned and unaligned sequences.

For very large numbers of sequences the distance matrix becomes very time and memory consuming to create. mBed [14] allows us to calculate approximate mean distances for the sequences, without calculating a full distance matrix. For analysing the vector of distances, we predict outliers in two ways: bootstrapping to estimate confidence intervals or by analysing interquartile ranges. Both of these methods are used to find sequences with unexpectedly large average distances.

#### Alignment gap metric

#### Aligned sequences

OD-Seq can read distance matrices in Phylip [15] format, unaligned sequences in Fasta format and alignment files in many different formats. If the sequences are in a multiple alignment, the alignment gap metric is used to measure how similar gap placement is between every pair of sequences and detects sequences with missing parts compared to the rest of the alignment. This happens with fragments, for example. It is used to produce a distance matrix by pairwise calculation of the distances in the alignment *X*_*N*,*L*_, with *N* number of sequences and *L* is the length of the alignment. Three different metrics are available for selection: linear, affine and cumulative. Before the distance is computed the sequences are changed to a binary representation with 0 for an amino acid character and 1 for a gap. The linear metric is given by the formula

(1) $$ S_{i,j} = \sum_{l=0}^{L} \left\{ \begin{array}{l l} 0, \quad X_{i,l} = X_{j,l} \\ 1, \quad X_{i,l} \neq X_{j,l} \end{array} \right.  $$

with distance matrix *S*, length of alignment *L* and the alignment as a matrix *X* in binary form. It does not distinguish between fewer, longer gaps or more, shorter gaps. The affine version uses the formula

(2) $$ S_{i,j} = \sum_{l=0}^{L} \left\{ \begin{array}{l l} 0, \quad X_{i,l} = X_{j,l}\\ 3, \quad X_{i,l} \neq X_{j,l} \: \& \: X_{i,l-1} = X_{j,l-1}\\ 1, \quad X_{i,l} \neq X_{j,l} \: \& \: X_{i,l-1} \neq X_{j,l-1} \end{array} \right.  $$

which scores short gaps relatively high compared to long ones, as it has an initial gap opening penalty. If the cumulative metric is chosen gap opening produces a low score but as the gap gets longer the extension score increases, the formula for this is

(3) $$ S_{i,j} = \sum_{l=0}^{L} \left\{ \begin{array}{l l} 0, \: \quad \quad \quad \quad X_{i,l} = X_{j,l}\\ 1, \: \quad \quad \quad \quad X_{i,0} \neq X_{j,0}\\ 1 + C_{l-1}, \quad X_{i,l} \neq X_{j,l} \: | \: l > 0 \end{array} \right.  $$

with *C*_*j*_ being a vector holding the distance value for each position of the comparison. The pairwise scores of each sequence M are then added up to an overall distance vector *D*_*i*_.

(4) $$ D_{i} = \sum_{j=0}^{M} S_{i,j}  $$

#### Unaligned sequences

The similarity computation for unaligned sequences is performed by BLASTP with default parameters. Here the bit score for every pairwise computation is added up to an overall score for each sequence.

#### mBed

Computing a full distance matrix for *N* sequences has a time and memory complexity of *O*(*N* ^2^). This becomes prohibitive for alignments of many sequences. The mBed algorithm, also used in Clustal Omega, reduces computing times. Here the *N*×*N* distance matrix is reduced to *N* log(*N*) by randomly selecting *M*= log(*N*) seed sequences and calculating a reduced *M*×*N* distance matrix. When tested on multiple sequence alignment benchmarks, it was used to make alignments without a significant change in accuracy.

#### Bootstrapping

To generate robust estimates of the mean and standard deviation of the distribution of the average distances, bootstrapping can be used. Pseudo replicates are created by picking *N* sequences randomly (with replacement) and computing the mean and standard deviation of each pseudo replicate. The mean of these values over all the replicates generate the estimates for the calculation of the outlier score. A sequence *i* is considered an outlier if

(5) $$ T < \frac{D_{i} - \overline{s}}{\sigma}  $$

with *σ* being the estimated standard deviation, $\overline {s}$ the estimated mean score and *D*_*i*_ the score of sequence *i*.

#### Interquartile range analysis

For the interquartile range analysis, the distance vector for the sequences is sorted. The values for the 1st quartile (*Q*1) and 3rd quartiles (*Q*3) determine the interquartile range *r*=*Q*3−*Q*1. For sequences with a distance measure greater than *Q*1 and smaller than *Q*3 the outlier score is 0. A sequence *i* is considered an outlier if

(6) $$ D_{i} < Q1 \quad \& \quad T > \frac {Q1 -D_{i}}{r}  $$

or

(7) $$ D_{i} > Q3 \quad \& \quad T < \frac{D_{i} - Q3}{r}  $$

Sequences are asigned a value of 0 and not considered an outlier if

(8) $$ D_{i} > Q1 \quad \& \quad D_{i} < Q3  $$

For datasets with a lot of identical sequences the interquartile range might be 0 which can cause a division by 0. Small values for the interquartile range can lead to inflated scores for the outliers. For these cases bootstrap analysis should be chosen as the estimators are more robust.

### Datasets

We used the Pfam database (version 27.0) of protein domains for all examples in this paper. The ParG family (PF09274) from Pfam is used as an example to illustrate the computation, it contains 92 sequences with an alignment length of 131. Otherwise, we take full alignments of Pfam domains and test our ability to detect outliers when sequences are added from other Pfam families. To introduce outliers, each dataset was realigned, including the outlier sequences, using Clustal Omega. Three different types of datasets were analysed. The first used all families with 5000 or more sequences. Here, 5000 sequences were randomly selected and 51 sequences added from a different family in the same Pfam clan. The second had 51 sequences added from randomly selected clans. The third is a control consisting of the original alignments, with no outliers, realigned with Clustal Omega. These contain 888, 1328 and 1328 alignments respectively (Table 1). To avoid artifacts caused by unequal sequence lengths, the outliers had to be within ±10 *%* of the average alignment length.

**Table 1:** Pfam verification datasets overview

	Different clan	Same clan	No outlier
Number of families	1329	889	1329
Number of sequences	5051	5051	5000
Average alignment length	906.7	817.4	664.3
% identity	27.5	25.1	29.0

Computing times were determined using two variations of the ABC transporter family in Pfam (PF00005). For measuring the time with various alignment lengths, 5000 sequences were chosen and truncated to various lengths. The dependency on the number of sequences in the alignment was determined by keeping the length constant at 200 positions and varying the number of sequences.

## Results

### Example analysis

**Fig. 2:**
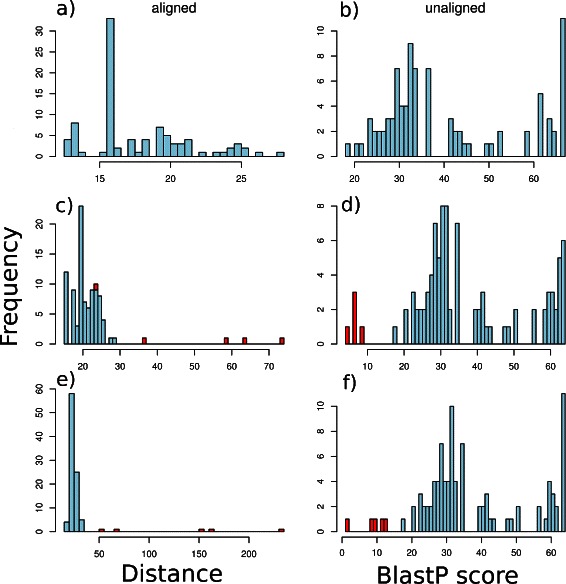
Example analysis unnormalised. Histograms of distance or similarity score distributions, using the ParG family from Pfam. **a**, **b**: Distribution with no outliers. **c**, **d**: Distribution with 5 outliers included from the same Pfam clan. **e**, **f**: Distribution with 5 outliers included from a different Pfam clan. The outliers are shown in red

We show one example of detecting outliers in detail with the ParG family from Pfam. This family has 92 sequences and the distance matrix has been calculated in full distance matrix mode. The distribution of average between sequence distances in the Pfam alignment is shown in the top left hand panel of Fig. 2. The left hand side of the figure shows results for aligned sequences in a multiple alignment calculated with the linear gap metric. The right hand panel shows the equivalent scores using BLASTP to measure between sequence similarity in the same unaligned sequences. In the second row of Fig. 2, the distances (on the left) and BLASTP scores (on the right) are shown after 5 random outlier sequences from the same Pfam clan as ParG (Met_repress, CL0057) have been artificially added to the datasets. In the bottom row of the figure, the distances are shown after adding 5 random outliers from random clans. We can clearly see that the average distances (or BLASTP scores) of the outliers to the rest of the sequences, make them reasonably distinct, especially in the case of outliers chosen from different clans.

**Fig. 3:**
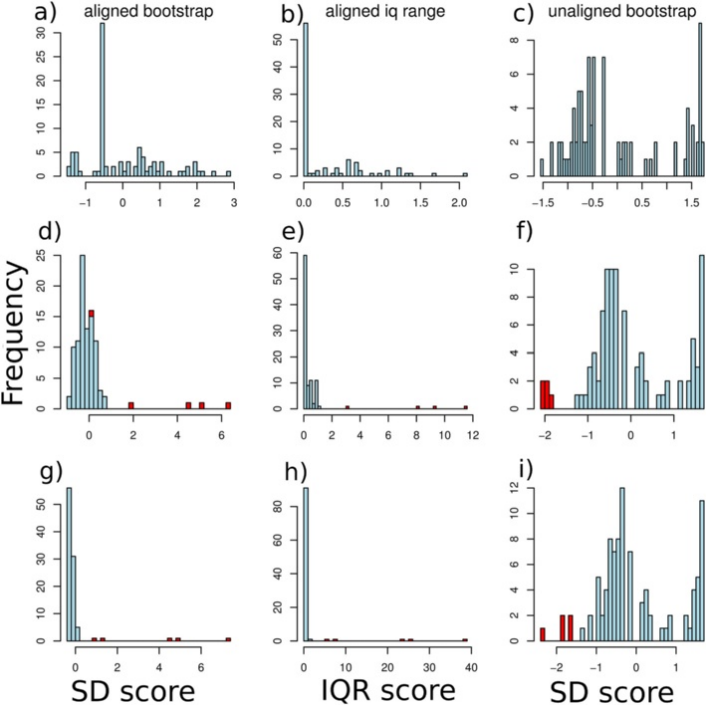
Example analysis normalised. Histograms of normalised distance (bootstrap and interquartile range) or similarity score (unaligned) distributions, using the ParG family from Pfam. **a**, **b**, **c**: Distribution with no outliers. **d**, **e**, **f**: Distribution with 5 outliers included from the same Pfam clan. **g**, **h**, **i**: Distribution with 5 outliers included from a different Pfam clan. The outliers are shown in red

The question is how to choose a cut-off that will allow us to try to automatically detect as many outliers with as few false positive sequences as possible, in other data sets. This is hard to do with the raw distances as these will depend on the particular set of sequences. The distances need to be normalised and this is done in two ways: distance standard deviations, estimated using the bootstrap or by taking interquartile ranges. The results, using these are shown in Fig. 3. Here, the left hand column shows bootstrapped standard deviations for the case with no outliers (top), outliers from the same clan (middle) or different clans (bottom). The middle column is the equivalent but here the horizontal axis shows interquartile ranges. The right hand column shows the BLASTP distances, normalised using bootstrap.

### Run times

**Fig. 4:**
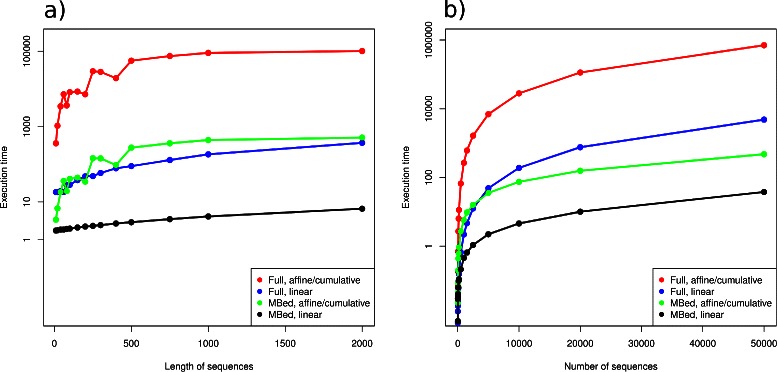
Computing times. Plot of compute times for varying length and number of sequences in mBed and full distance mode for the 3 different metrics. The execution times are measured in seconds and shown on the y-axis. In **a** the datasets contain 5000 sequences with length varying from 10 to 2000 amino acids (x-axis). The datasets used in **b** consist of 10 to 50,000 sequences (x-axis) of length 200

The run times for the algorithm are expected to show a minimum complexity of *O*(*L*) in sequence length. Times for different subsets of the ABC transporters of different lengths are plotted in (Fig. 4 a) and all variations of the algorithm do show approximately linear behaviour. Time complexity wrt number of sequences is expected to be *O*(*N* ^2^) for full distance matrix mode or *O*(*N* log(*N*)) for mBed mode. Run times for different subsets of the ABC transporters are plotted in (Fig. 4 b). Data shown is the execution time for the whole analysis including sequence read, distance computation, normalisation and output. The outlier detection for the whole ABC transporter family from Pfam with 363,409 sequences with an alignment length of 2177 takes 39 minutes on one CPU core.

### Large scale outlier tests

**Fig. 5:**
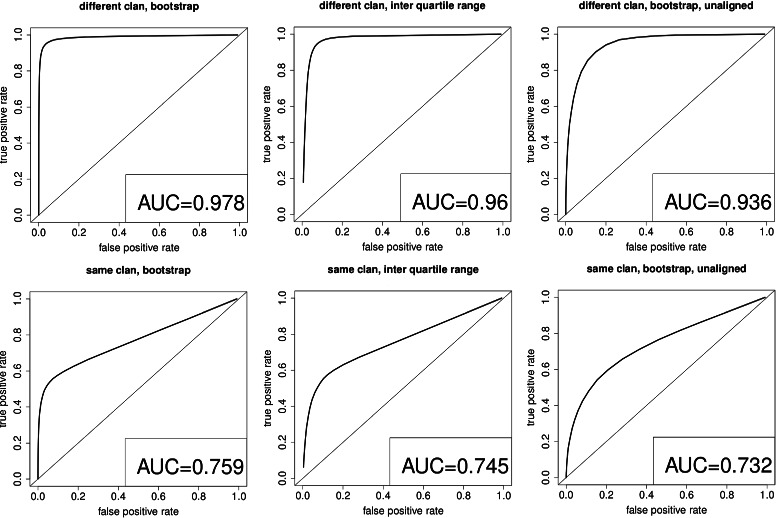
Receiver operating characteristic curves. ROC curves to verify the detectability of artificially introduced outliers in Pfam families (Table 1). It shows true and false positive values for different cutoffs with AUC of 0.978, 0.96 and 0.936 for datasets where outliers from different clans have been introduced. When introduced outliers are from the same clan AUC’s of 0.759, 0.745 and 0.732 are shown. The diagonal line represents random classification

**Fig. 6:**
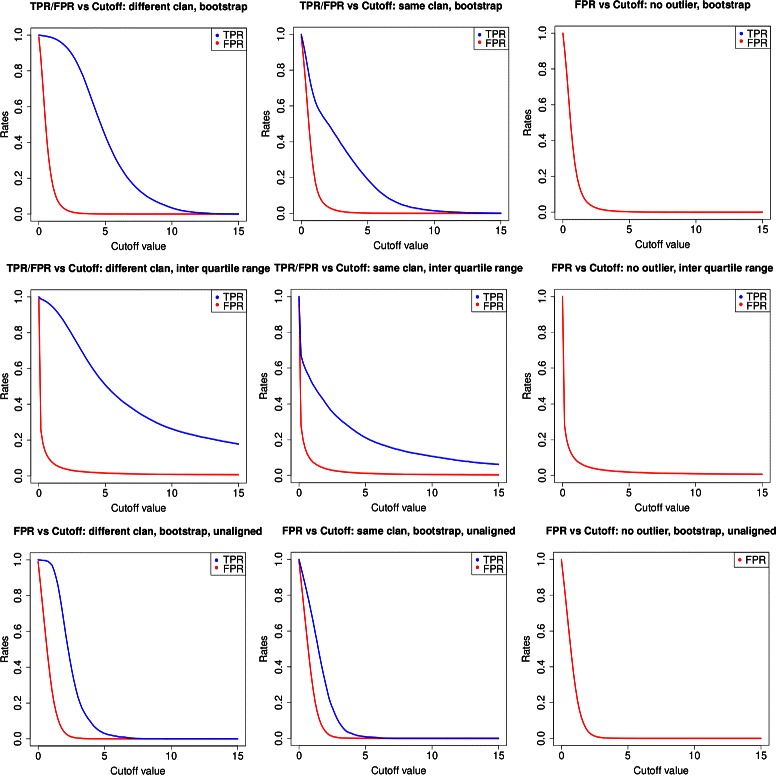
True and false positive rates against cutoff. The true and false positive rates are plotted against the cutoff to observe suitable cutoff values on the x-axis. True positive rates are shown in blue and false positive rates in red on the y-axis. The columns show the different datasets: outliers from an unrelated Pfam clan, outliers from the same clan and no outliers respectively. Rows vary the method of normalisation (bootstrap, interquartile range and unaligned bootstrap)

The overall performance of OD-seq when tested on over 1000 Pfam families, seeded with outliers from the same, or from a different clan, are summarised in Fig. 5. Here we use receiver operating characteristic (ROC) curves to show the trade-off between sensitivity and specificity for different cut-offs and different OD-seq options. When outliers from a different clan are used, the ability of OD-seq to detect them accurately, with low false positive rates, can be seen with area under the curve (AUC) values of well over 0.9. The bootstrap normalised distances (top left panel) do slightly better than the interquartile range ones (0.98 vs. 096) with the aligned sequences and the unaligned sequences do slightly worse with an AUC of 0.93. The lower 3 panels of Fig. 6 show the ROC curves when outliers are seeded from the same clan. This is a much harder test but AUCs of 0.73–0.76 are still achieved.

A threshold range for outliers was identified by plotting the true positive (TPR) and false positive (FPR) rate against the threshold (Fig. 5). In these plots one can observe that if a cut-off of 2 is selected a FPR below 10 *%* can be expected while the TPR is still far higher and depending on the dataset true outliers can be identified confidently. The plots show that the FPR stays constant even if no outliers are present. According to these figures a threshold between 2 and 10 is recommended, depending on the stringency the user wants to choose. For the same clan datasets a steep decline in TPR is observed, this is due to the fact that many of the introduced outliers are so similar to the selected family that they might not even be considered outliers due to their similarity. For the unaligned method the area of good performance is narrower as high outlier scores are very rare.

**Fig. 7:**
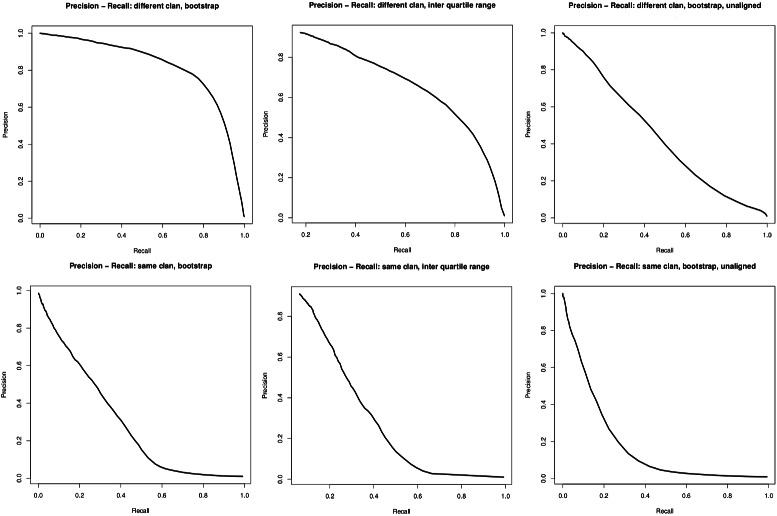
Precision recall curves. The precision recall curves plot precision on the y-axis against recall values on the x-axis. The first row shows the Pfam datasets with outliers from different clans, the second row shows datasets from Pfam with outliers from the same clan. The columns show the different methods of normalization: aligned bootstrap, interquartile range and unaligned bootstrap respectively

The ROC curves and TPR/FPR plots show that finding outliers is possible with OD-seq, but as the ratio between outliers and core sequences is 100:1 missing an outlier is punished strongly and misclassifying a lot of core sequences as outliers do not increase the FPR by much. To assess the differences between different modes and metrics of OD-seq these plots do not give a lot of information. Therefore precision recall curves, which scale the TPR and FPR by the number of outliers, were produced. This makes finding outliers and core sequences comparable (Fig. 7). Here one can clearly see that the use of a multiple sequence alignment enhances outlier detection. The linear metric with bootstrap analysis performs best as the prediction is more robust than with the interquartile range analysis.

### Pfam outliers and example

Table 2 shows the percentage of outliers predicted by OD-seq for full Pfam families of different sizes (number of sequences in the family) for 10 different cutoffs and 4 modes.

**Table 2:** Detected outliers in Pfam families

Pfam families			Percentage of outliers found by bootstrap cutoff							
Sequences	Median	Type	1	2	3	4	5	6	7	8
≤150	68	Linear	12.52	5.51	2.22	0.92	0.40	0.19	0.09	0.05
150−500	278		12.70	5.34	2.11	0.88	0.39	0.19	0.09	0.05
≥500	1652		11.74	5.21	2.25	1.00	0.46	0.22	0.11	0.06
≤150	68	Affine	13.10	5.39	2.02	0.76	0.33	0.15	0.07	0.03
150−500	278		13.25	5.33	2.00	0.77	0.32	0.14	0.07	0.03
≥500	1652		11.98	5.18	2.21	0.96	0.43	0.19	0.09	0.05
≤150	68	Cumulative	10.37	5.10	2.54	1.30	0.69	0.40	0.22	0.12
150−500	278		9.01	4.25	2.15	1.20	0.72	0.45	0.29	0.20
≥500	1652		7.50	3.53	1.87	1.09	0.68	0.45	0.31	0.22
≤150	68	Unaligned	14.3	8.19	1.01	0.055	0.046	0.045	0.045	0.045
150−500	278		16.35	12.65	0.093	0.012	0.0027	0.0009	0.0005	0.0003
≥500	1652		15.8	1.65	0.16	0.018	0.0032	0.0008	0.0002	0.0001

**Fig. 8:**
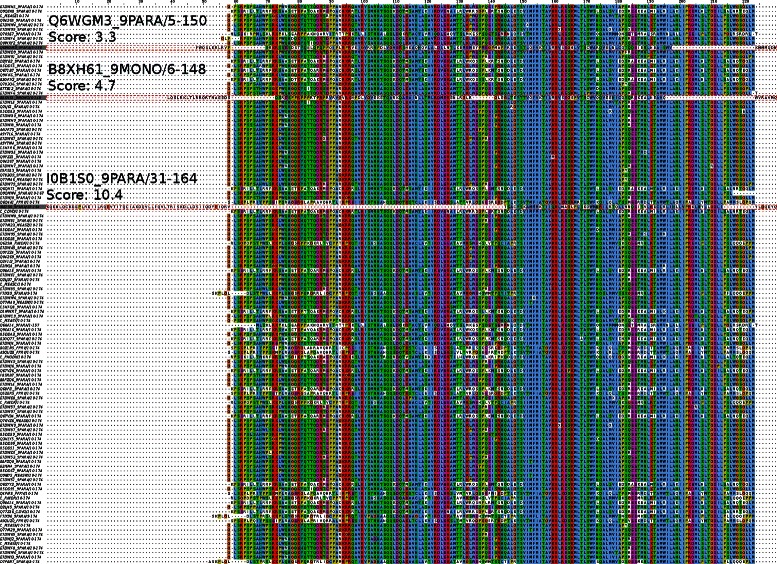
Outlier example. Alignment of sequences of the Pfam family Paramyxo_NS_C (non-structural protein C). 3 sequences with high outlier scores are highlighted (I0B1S0_9PARA/31-164, B8XH61_0MONO/6-148 and Q6WGM3_9PARA/5-150), the scores are 10.4, 4.7 and 3.3 respectively

In this example we take the non-structural protein C (Paramyxo_NS_C) family from Pfam with 123 sequences and an alignment length of 230 amino acids and show it with Jalview. In this typical example alignment three outliers were identified (Fig. 8). One can see that the most obvious outlier (I0B1S0_9PARA/31-164) misaligned with almost all residues except a small core region (Outlier score of 10.4). the same happens for the other two sequences with a smaller but detectable misalignment (Outlier score 4.7 for B8XH61_0MONO/6-148 and 3.3 for Q6WGM3_9PARA/5-150).

## Discussion

Viewing very large alignments of many thousands of sequences is challenging, even if you use the best viewers available such as Jalview or Seaview [16]. One serious issue is to detect and deal with highly aberrant sequences. OD-seq provides a simple yet robust method to rank the most aberrant sequences or to select all sequences above some cut-off that is tuned to optimise a desired false positive or false negative rate. When faced with clear outliers, such as the case with Pfam families that have been seeded with sequences from different Pfam clans, OD-seq finds the outliers with extremely high sensitivity and specificity. These outliers were selected to be similar in length to the family they were added to so as to reduce length effects. When sequences were added from the same Pfam clan, the performance reduces but here, the difference between an outlier and a homologous yet very dissimilar sequence, is not so clear-cut. OD-seq can also deal with unaligned sequences, but the performance is, again, not as good as the full multiple alignment case. The main aim when designing OD-seq was to catch outliers in the full alignment.

OD-seq is fast enough to process alignments of moderate to large size in seconds. The run times to do any calculations on a full distance matrix will normally be proportional to the square of the number of sequences. For alignments of many tens of thousands of sequences this could take days to process. The *O*(*N* log(*N*)) mBed method for distance matrix calculation, reduces the time and memory to perfectly practical levels with alignments of over 300,000 sequences of length over 2000 positions being processed in under 40 minutes on a single core. The combination of speed and accuracy, then makes OD-seq a potentially useful tool for pipelines that require many large alignments to be made or for checking very large alignments for outliers. OD-seq will not be able to deal with outliers in very small data sets or with sequences that are homologous to the rest of a dataset over most of their length but which have a short region of mismatch. Such sequences or mismatched regions can be found satisfactorily by existing tools.

## Conclusion

OD-seq is a practical and simple method to detect outliers in MSAs. It can also detect outliers in sets of unaligned sequences, but with reduced accuracy. For medium sized alignments, of a few thousand sequences, it can detect outliers in a few seconds.
